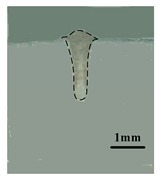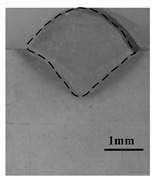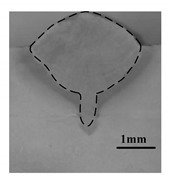# Physical Characteristics of Coupled Plasma and Its Influence on Weld Formation in Hybrid Laser-Double-Arc Welding

**DOI:** 10.3390/ma12244207

**Published:** 2019-12-14

**Authors:** Xiaoyan Gu, Yuchen Liu, Wenhang Li, Yujun Han, Kai Zheng

**Affiliations:** 1 Jiangsu Key Laboratory of Advanced Welding Technology, Jiangsu University of Science and Technology, Zhenjiang 212003, China; liligu1983@163.com (X.G.); liuyuchen123@163.com (Y.L.); 2 School of Materials Science and Engineering, Nanjing Institute of Technology, Nanjing 211167, China; yujun.han@mail.wvu.edu; 3 Research and Development Center, CRRC Qingdao Sifang Rolling Stock Research Institute Co., Ltd, Qingdao 266031, China; zheng_kai@tju.edu.cn

**Keywords:** hybrid laser-double-arc welding, coupled plasma, electron temperature, electron density, arc shape

## Abstract

Hybrid laser-double-arc welding (HLDAW) is an efficient welding method with multi-heat sources comprised of two arcs and a laser beam, which is an intricate process with much randomness and uncertainty due to the mutual effect between multi-plasmas. Compared with double-arc welding (DAW), HLDAW can generally obtain a more stable welding process and deeper weld penetration, which is directly affected by the synergistic effect of multiple heat sources; however, the mechanism has not been systematically studied. In this study, the multi-information analysis method is adopted to study the distribution of electron temperatures, electron densities and electrical conductivity of double-arc welding (DAW) and HLDAW by utilizing synchronous radiation spectrum, high-speed photography and electrical signal sensing technology. The results indicated that the high concentration of charged particles provided a conductive channel for the two arcs to achieve a more stable welding process in HLDAW. The synergy between the laser and the arc changed the flow mode of the weld pool, which facilitated the molten metal flowing toward the bottom of the molten pool. Obtaining the same melting depth, the line energy input of HLDAW was 13% lower than that of DAW; the heat affected zone is narrower and the grain size is smaller. The weld penetration depth was improved in HLDAW, which was 1.8 times that of DAW and 1.5 times that of pure laser welding under the laser power of 1.5 kW. The weld penetration of HLDAW increased with laser power.

## 1. Introduction

Hybrid laser-double-arc welding (HLDAW) is a novel welding method based on double-arc welding and laser welding, and compared with hybrid laser-single-arc welding, this welding method can achieve higher welding speed, lower line energy and the gap allowance is up to 2 mm [1,2,3]. In addition, the welding process is more stable than double arc welding [4]. Moreover, this kind of welding equipment is free from the limitation of welding direction, and the arc space can be adjusted according to application, which can better realize automatic welding [5,6]. It has a great application prospect in welding low carbon steel, high strength steel and stainless steel, especially for the workpiece with a thickness of more than 20 mm.

However, compared to the hybrid laser single arc welding and arc welding, it is an intricate process with many randomness and uncertainties due to the mutual effect between triple heat sources [7,8], which restricted its application.

At present, the research on HLDAW is less, and mainly focuses on the process exploration. Hu [9] analyzed the influence of the distance between two arcs on plasma interaction and droplet transfer through a high speed camera system and an electrical signal acquisition system. Wei et al. [4,10,11] qualitatively discussed the coupling mechanism of laser beam and arcs through an observation of electrical signals and high-speed camera signals in HLDAW. Gu et al. [12] calculated the largest Lyapunov exponent (LLE) of characteristic currents during the welding process under different arc distance by the phase space reconstruction technique to evaluate the stability of HLDAW. Gu et al. [13] discovered a special phenomenon alternative burning behavior of arcs in HLDAW and explored the change laws of alternative burning and its mechanism. The research results indicated that a certain synergy effect between laser and arcs brings benefits, and in some cases it is difficult to achieve better welding results [1,14,15,16,17]. A comprehensive and profound understanding of the synergy effect between laser and arcs will provides higher utilization of HLDAW in industry. Quantitative analysis of the synergy effect and its influences on the welding process are of great significance for optimizing welding quality.

In this paper, we examined electron temperatures, electron densities and electrical conductivity for coupled plasma of HLDAW using optical emission spectroscopy to better understand the physical characteristics of coupled plasma. Its influence on weld forming and penetration depth was further analyzed, meanwhile synchronous high-speed photography and electrical signal were adopted to observe arc shape to reveal the physical principle of the synergy effect in order to find the fundamental mechanism of HLDAW.

## 2. Materials and Methods

In this study, Q235B mild steel plate (Taiyuan Iron & Steel (Group) CO., LTD, Taiyuan, China) with the dimensions of 310 mm × 150 mm × 12 mm was used as a base metal, while H08Mn2SiA copper (Sichuan Atlantic welding materials CO., LTD, Zigong, China)-coated wire with a diameter of 1.2 mm was used as filler material. The composition of the Q235B mild steel and the welding wire are shown in Table 1. Surfacing was adopted in this experiment. Argon was used as shielding gas with flow rate of 20 L·min^−1^.

**Figure 1:**
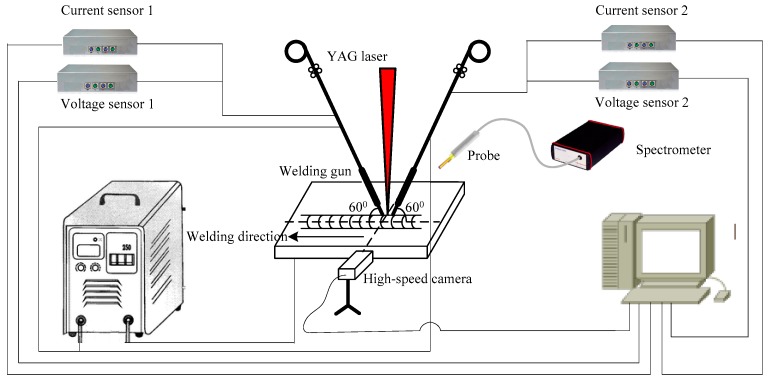
Schematic of the hybrid laser-double-arc welding (HLDAW) system.

The schematic illustration of the HLDAW system is shown in Figure 1. The welding machine employed in the experiment was composed of a ND: YAG laser (JK2003SM, GSI Group Laser Div. Rugby, England) with a maximum rated power of 2000 W. A Lincon INVERTEC V350-PRO (Lincoln welding machine (Shanghai) CO., LTD, Shanghai, China) power source and supporting wire feeder were used along with the laser device to achieve a hybrid welding process. A continuous wave ND: YAG laser beam of wavelength 1064 nm was concentrated on the surface of the workpiece with a spot radius of 80 μm and the focal length of 300 mm. The arc welding guns exist in same plane of weld bead. The angle between the central axes of these welding guns and our workpiece were kept at 60°. The laser beam was located in the same plane with the two welding guns, and the angle between them was 30°. The defocusing amount kept at 0 mm, the distance between laser beam and welding tip was 3.5 mm, the welding speed was kept 5 mm/s and the wire feed rate was 2.5 m/min. There are 22 specimens prepared by pure laser, DAW and HLDAW, the specific welding parameters for every specimen are shown in Table 2.

The welding monitoring system adopted synchronous multi-information fusion technology, which comprised an electrical signal sensor device, (PCI-1742 DAQ Card, Acitetch- Newcite Information Technology Co.,Ltd, Shanghai, China) with the frequency of 10 kHz, a high-speed photography device (RockeTech technology Corp., Ltd, Changsha, China, CPL 250 K CMOS) with the sampling frequency of 1000 frames/s) for recording droplet transfer and welding plasma morphology and an optical detection device for detecting the light emission of hybrid plasma. A fiber optic spectrometer (AvaSpec-2048, Avantes Technology Co.,Ltd, Beijing, China) with wavelength range of 200–1100 nm and sampling precision of 0.09 nm was used as the detection device to detect the light emission of hybrid plasma. Specific welding parameters are shown in Table 1.

**Figure 2:**
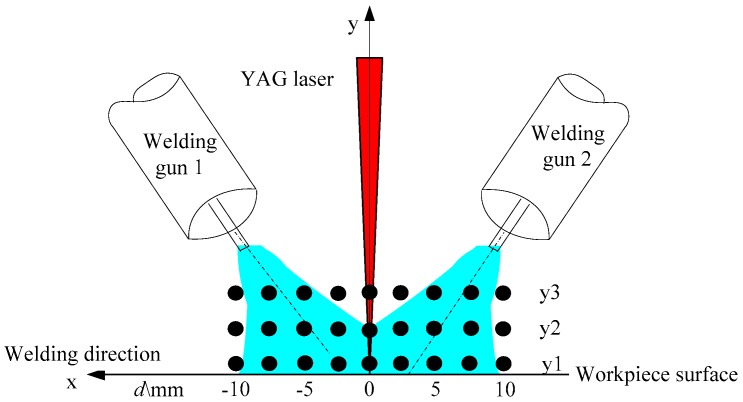
Measurement points distribution.

In this experiment, a long, thin, hollow tube with a diameter of 1.0 mm was mounted towards the probe to collect the light emitted from the hybrid plasma. In this way, the spatial location of the collection point can be obtained accurately. In addition, the thin tube limits the amount of light passing through, resulting in a significant reduction in radiation intensity, which is effective for resolving the problems of radiation saturation and spectrograph protection. The measurement point distribution was shown in Figure 2, y1 is 0.5 mm above the plate surface, y2 is 2.0 mm above the plate surface and the top layer y3 is 6 mm above the plate surface. In this experiment, it was easily to obtain the spectral radiation intensity at the peak pulse, because the three kinds of signals including electrical signals, high-speed camera signals and spectral signals, were synchronized. To ensure accuracy, each point is measured three times, and the average value is taken as the radiation intensity at this point.

## 3. Results and Discussion

It is assumed that hybrid plasma of HLDAW should be optically thin and in local thermal equilibrium (LTE). By this assumption, the distribution of energy in the plasma region follows Maxwell’s equation [18]. The LTE assumption can be satisfied due to sufficiently high electron density [19]:

(1) $$N_{e}\geq 1.6\times 10^{12}T_{e}^{1/2}{\left(\Delta E\right)}^{3}\left(cm^{-3}\right)$$

where $N_{e}$ is the electron density, $T_{e}$ is the electron temperature, and ΔE is the energy gap.

The maximum electron temperature of HLDAW plasma is below 20,000 K, the maximum ΔE of the selected FeI feature lines is 3.0646 eV. According to Equation (1), the minimum electron density of the welding plasma satisfying the local thermodynamic equilibrium condition is 6.45 × 10^15^ cm^−3^.

The intensity of a spectral line emitted characteristic line satisfies the expression as follows when atoms transit from higher energy m to lower energy n [20]:

(2) $$\ln\left(\frac{I_{mn}}{\nu_{mn}g_{m}A_{mn}}\right)=-\frac{{Ε}_{m}}{\kappa {Τ}_{e}}+D$$

In which, D is constant, $A_{mn}$ is the transition probability, $g_{m}$ is the statistic-weight factor, ${Ε}_{m}$ is the energy in the higher level, $I_{mn}$ is the radiation intensity, $\nu_{mn}$ is the radiation frequency and κ is the Boltzmann constant. In Equation (2), ${Ε}_{m}$ is set as *X*-axis and $\ln\left(I_{mn}/\nu_{mn}g_{m}A_{mn}\right)$ as *Y*-axis.

The specific method is selecting several line spectra of the same element and obtaining the corresponding point plotted on the XOY plane coordinates through every selected line spectrum by Equation (2). After fitting points linearly, the electron temperature ${Τ}_{e}$ can be obtained from its slope.

**Figure 3:**
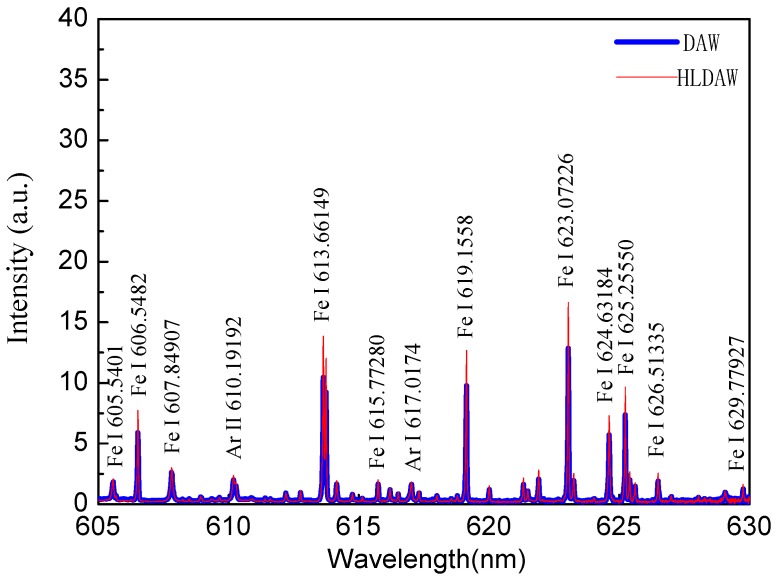
Calibration of feature spectrum line between 605–630 nm.

Figure 3 is the calibration of the feature spectrum line between 605–630 nm. In this paper, four FeI lines (629.77927 nm, 617.33352 nm, 600.30119 nm and 606.5482 nm) were chosen as characteristic lines to calculate the electron temperature of hybrid plasma on different layers. The parameters of the feature spectrum lines are shown in Table 3.

The electron density of arc plasma can be acquired from Equation (3) [21]:

(3) $$N_{e}=\frac{\Delta \lambda_{1/2}^{S}}{2\omega }\Delta 10^{16}$$

In which, $\Delta \lambda_{1/2}^{S}$ is full width at half maximum, ω is coefficient of electron collision–broadening, which can be obtained from [21,22].

**Figure 4:**
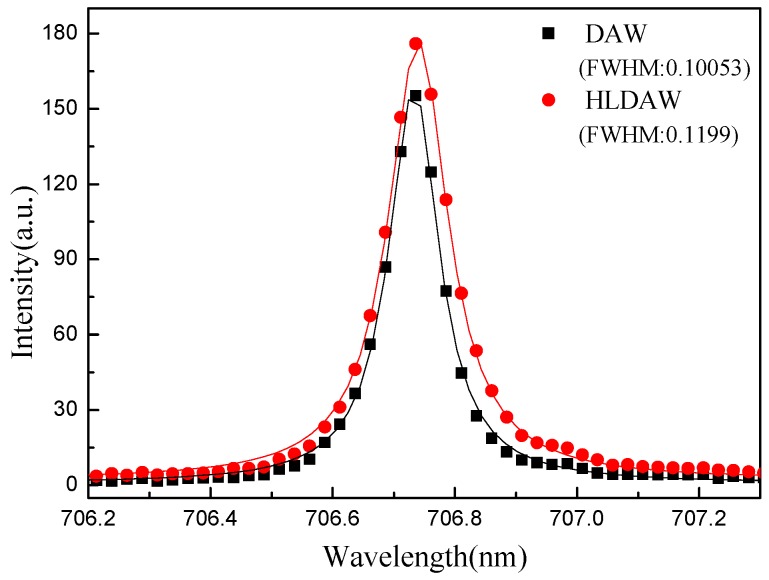
Lorentzian fitting of the Stark broadened profile for ArI 706.7218 nm.

Spectral lines with appropriate intensity and good separability were selected and fitted linearly by Lorentz to obtain $\Delta \lambda_{1/2}^{S}$. In this research, ArI 706.7218 was selected as a characteristic spectral line to calculate $N_{e}$. The fitting results were shown in Figure 4.

The electron temperature of the hybrid plasma calculated from Equation (2) ranges from 12,000 to 17,000 K. According to [21], the value of ω is 0.0066 nm.

**Figure 5:**
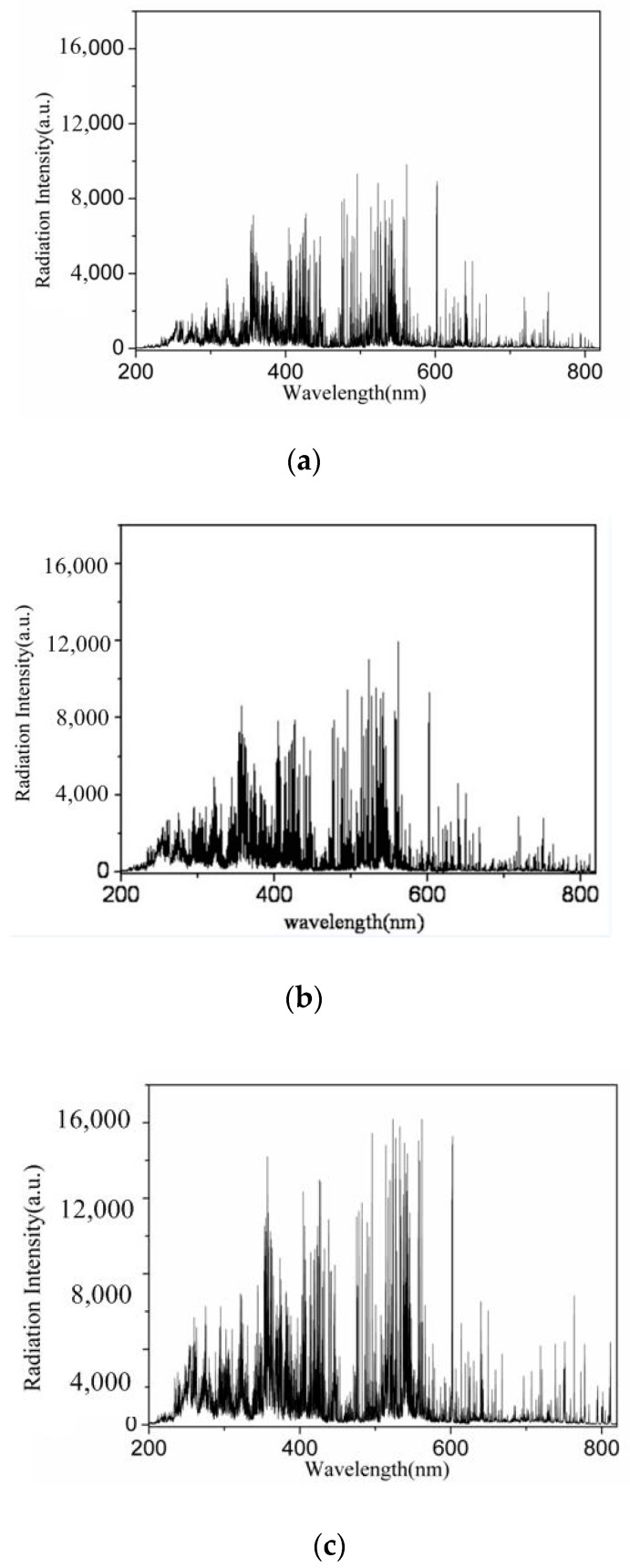
Radiation comparison of laser point in different laser powers. (**a**) without laser (**b**) with 500 W laser (**c**) with 1.5 kW laser.

Figure 5 shows the continuous spectral radiation of the midpoint of two arcs on layer y1 in different laser powers. Figure 5a is the radiation intensity of DAW, while b and c are the radiation intensity after 0.5 kW and 1.5 kW laser addition, respectively.

It can be obtained from Figure 5 that the radiation spectrum of hybrid plasma was basically the same as that before the laser addition, where there were a large number of lines distributing on a continuous spectrum. The line spectrum was composed of FeⅠ, FeⅡ ranging from 200~350 nm, FeⅠ ranging from 350~690 nm and ArⅠ ranging from 690~820 nm. However, the radiation intensity of HLDAW was stronger than that of DAW, especially in the UV-visible region that is mainly dominated by iron particles. In addition, the radiation intensity increased with laser power. It means that the introduction of laser could not bring new radiation lines, but increase the degree of particle ionization, and this enhanced effect improved with the increase of laser power.

**Figure 6:**
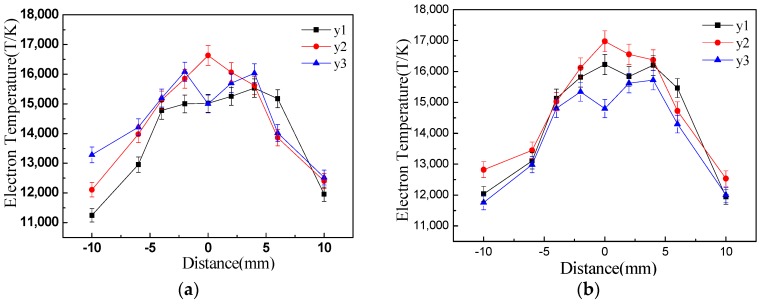
Distribution of electron temperature. (**a**) double-arc welding (DAW) (**b**) hybrid laser-double-arc welding (HLDAW) (P = 1.5 kW).

Figure 6 is the electron temperature distribution of different layers on peak pulse current with and without laser addition. It can be seen that the variation trends of electron temperature were almost the same with and without laser addition. For DAW, the temperature of the region near the midpoint of the two arcs (−5~5 mm) was higher than that of the region far away from the midpoint of the two arcs on the lower surface. The overall temperature distribution in middle layer (y2) was greater than the lower layer, and the zone with highest temperature and great gradient was formed on the midpoint of the two arcs, which was about 16,800 K. With further increase of the distance from the plate (y3), the temperature on the midpoint of the two arcs significantly decreased to about 15,000 K, the temperature peak on this layer was in the arc center, which was about 16,200 K, and the temperature distribution of other region was almost the same with y2.

For HLDAW, temperature distribution was similar with that of DAW. The temperature on the lower layer was about 1000 K higher than that of DAW. The highest temperature was also on the mid-tier layer (y2), which was about 17,200 K and 400 K higher than DAW. However, the overall temperature on y3 declined dramatically, even below that of y1.

The above calculation of electron temperature shows that the energy of HLDAW heat system increased, especially in the region near the weld pool.

**Figure 7:**
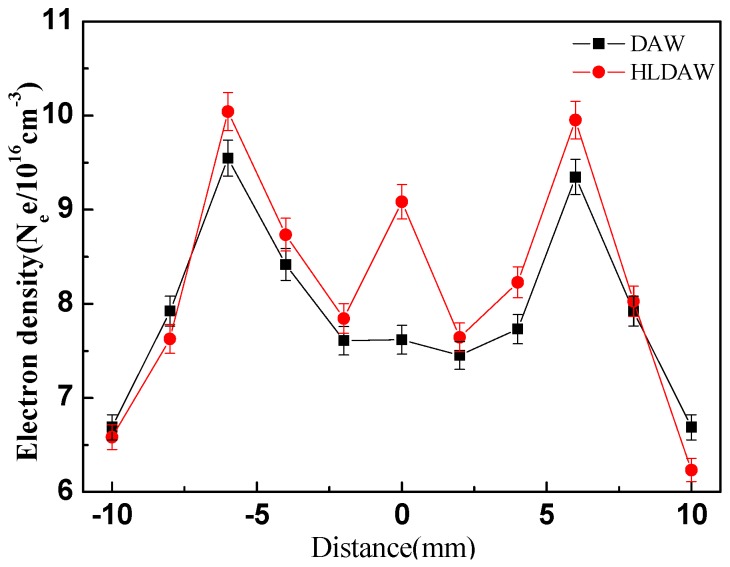
Distribution of electron density (layer 1).

Figure 7 shows the electron density distribution on a region close to molten pool (layer y1). It can be seen that the electron density of DAW and HLDAW presented a similar distribution trend, the peak regions were both in the center of arc column, while the electron density is lower in the regions that far away from the arc column. The electron density distribution of HLDAW is similar with DAW, but it is larger than that of DAW as a whole. The electron density mutated in the laser point, which is 1.25 times that of DAW.

**Figure 8:**
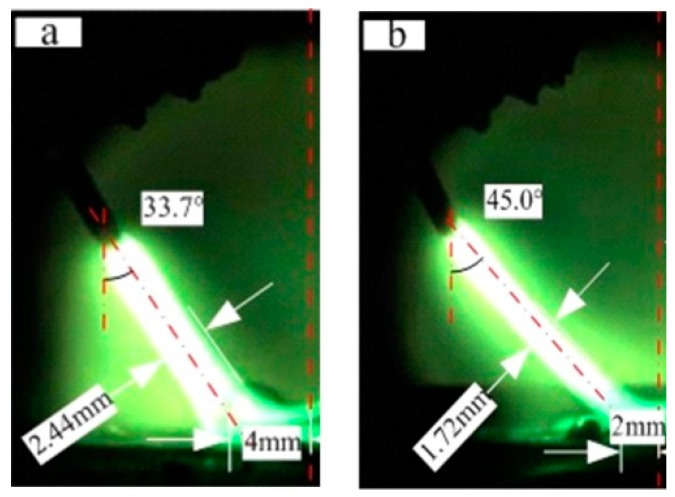
Arc shape of DAW and HLDAW. (**a**) Without laser (**b**) with 1.5 kW laser.

Figure 8 provides direct photographic evidence for the results above. In order to be more intuitive, the left arc in the two welding processes was selected for a comparison. Figure 8 is the arc morphology of DAW (a) and HLDAW (b) of peak pulse current. It can be observed that the angle between vertical direction and the central line of the arc was 33.7°, the arc–column diameter was 2.44 mm, and half of the distance between the two arc-roots was about 8.0 mm in DAW.

However, after addition of the 1.5 kW laser, the angle between vertical direction and the central line of the arc increased to 45°, the arc–column diameter reduced to 1.72 mm and the half spacing between the two arc-roots shortened to about 4.0 mm.

It demonstrated that the laser beam was imposing contraction and attraction on arcs, which made the cross section of the arc column smaller and the arc-root closer to the laser spot impinging on the molten pool surface.

In the welding process, metal of droplet and molten pool evaporated and ionized by absorbing the arc energy to result in the increases of the concentration of iron ions in the arc atmosphere. Compared with Fe, the ionization potential of Ar in the shielding gas is relatively small. The concentration of Ar ions in the arc atmosphere is lower. So, compared with ArⅠ ranging from 650~1100 nm, the radiation intensity of FeⅠ ranging from 200~350 nm and 350~690 nm is stronger. It indicated that the energy input of this welding system increased after laser addition to lead to the ionization of metal and the increase of metal vapor concentration, and this enhanced effect increases with the increase of laser power.

During the welding process, both of the shielding gas and gasified workpiece material further ionized into electrons and charged particles by absorbing heats to result in the increases of concentration of charged ions in the arc atmosphere.

The ionic potential of iron atoms is 762.5 kJ mol^−1^, much lower than that of the argon, which is 1520.6 kJ mol^−1^. Therefore, the number of ions was significantly higher than that of argon ions, and that which was shown in the spectrogram was that the quantity of iron emission lines was larger than that of argon, and the radiation intensity was also stronger. In the HLDAW process, at the position where the laser spot impinges upon the workpiece surface, the material was obviously vaporized because of the absorption of laser energy, and then the steam transported into the arc plasma [23,24,25]. The ionic potential of the atmosphere around the arc is reduced effectively. In addition, some of the laser beam energy can be absorbed by the arc plasma, which will help to further ionize the arc plasma [26].

Compared with DAW, the radiation intensity of these charged particles was much stronger in HLDAW, especially in the regions that were occupied by iron ions.

It can be inferred from Figure 5 and Figure 6 that the electron temperature and electron density near the center line of two arcs (laser beam in HLDAW) on the lower layer were prominently increased after introduction of the laser into this welding system.

The conductivity of the coupled plasma can be represented as [27]:

(4) $$\sigma =\frac{n_{e}e^{2}}{m_{e}\nu_{ei}}$$

(5) νei=8π(e24πε0)2(nme12Te32Kb32)lnΛ

where $n_{e}$ is the electron density, $\nu_{ei}$ is the collisions frequency between electrons and neutral particle, e is the electron charge, $m_{e}$ is the electron mass, $K_{b}$ is Boltzmann’s constant, $T_{e}$ is electron temperature, lnΛ is the coulomb logarithm. It can be inferred from Equations (4) and (5) that the conductivity of the coupled plasma increased with the electron temperature and electron density. Thus, the high concentration of charged particles provided a conductive channel for the two arcs. According to the principle of minimum voltage, the arc will automatically select the combustion region with the least resistance. In this way, the arc roots were confined to the laser spot impinging on the workpiece surface, achieving a stable welding process.

**Figure 9:**
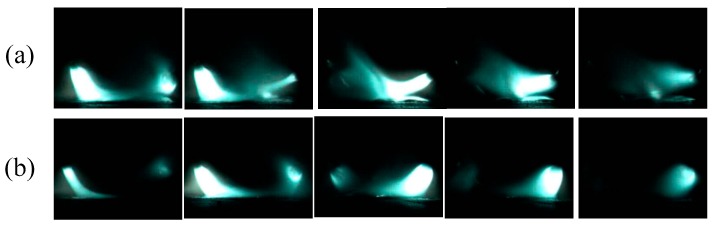
Dynamic process of arc. (**a**) DAW (**b**) HLDAW (P = 1.5 kW).

Figure 9a,b show the dynamic process of the arc within 15 ms at a welding speed of 8 mm/s during DAW and HLDAW, respectively. It demonstrated that the drifting of arc-root and severe bending of arc–column occurred as the electrode moving forward because of the mutual interference between the two arcs in DAW at a high welding speed. However, more stable cathode spots and smoother arc were obtained due to the conducting path near the laser point on the workpiece surface formed by the ionized particles, which makes the welding process stable after adding the 1.5 kW laser in HLDAW.

**Figure 10:**
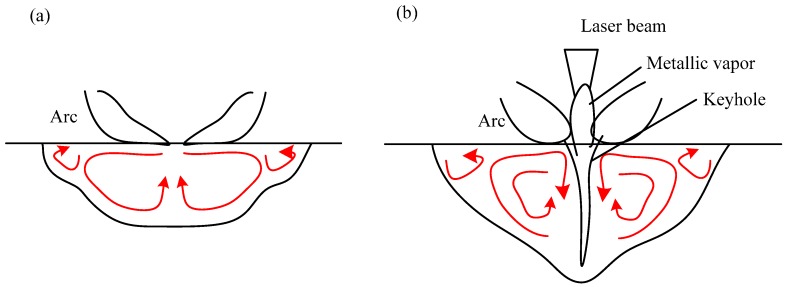
Comparison of liquid flow in molten pool. (**a**) DAW (**b**) HLDAW.

The material at the point of deposition rises in temperature to the boiling point, which converts liquid to superheated vapor. Once vapor is formed, it expands and is released upward from the surface, and produces a reaction force that presses the melt downward and sideways. The depressed surface of the weld pool permits additional photos from the laser beam and electrons/ions from the arc to impinge upon fresh material, which is then heated in the same way. In DAW, the driving force on the molten surface produced by two arcs pushing the melted metal flowing from the center to the edge of the weld pool is shown in Figure 10a. 

In the process of HLDAW, the liquid metal flow in the molten pool was different from that of DAW because of the addition of the laser, as shown in Figure 10b. The depression of the weld pool surface becomes larger because of the laser beam and transforms to a keyhole, the entire central core of which consists of vapor surrounded on all sides by an envelope of liquid metal [28]. The reaction force produced by vapor ejected upward from the keyhole facilitates the molten metal flowing toward the bottom of the molten pool. Meanwhile, the laser beam can pass through the keyhole and heat the bottom surface of penetration directly.

The drawing and compressing imposed by the laser beam on two arcs made the arc column shrink and increasing of electron density, which enhanced the Lorentz force in the molten pool. It increased the current density of the laser point location on the molten pool surface, promoting the liquid metal from the periphery of the weld pool to the center and then to the bottom.

In HLDAW, the surface deformation is the synthetical result caused by multiple forces including the arc pressure, the evaporation counterforce and the surface tension. The force on the free surface of the molten pool is expressed as follows:

(6) $$P=P_{A}+P_{R}-P_{S}$$

where $P_{A}$ is the arc pressure, $P_{R}$ is the evaporation counterforce and $P_{S}$ is the surface tension.

The compression of the arc column reduces the contact area between arc and liquid pool, which increases the arc blowing pressure on the pool and further helping the liquid metal to flow to the bottom.

In conclusion, the addition of the laser changed the flow of liquid metal in this HLDAW process. The flowing from the periphery to the center of the pool enhanced the heat transfer to the bottom of the pool, which was helpful for the high temperature and superheated liquid metal to move downward the bottom of the pool and increase the weld depth. The increases of welding penetration depth in HLDAW are also dependent largely upon the impact force of rapid-floating charged particles applied on the workpiece surface. Both of the number of charged particles and traveling speed are improved with the increase of laser intensity, thus increasing this impact force on the workpiece.

Table 4 shows the morphologies of the appearance and cross sections of the welding seam obtained by pure laser welding (2#), DAW(3#) and HLDAW(16#). Compared with pure laser welding and DAW, the weld penetration was deeper and the surface was more regular and smoother in HLDAW. It was found that the upper part of the weld penetration presented the characteristics of DAW, while the lower part presented the characteristics of pure laser welding. The weld depth is larger than that of pure laser welding and DAW.

**Figure 11:**
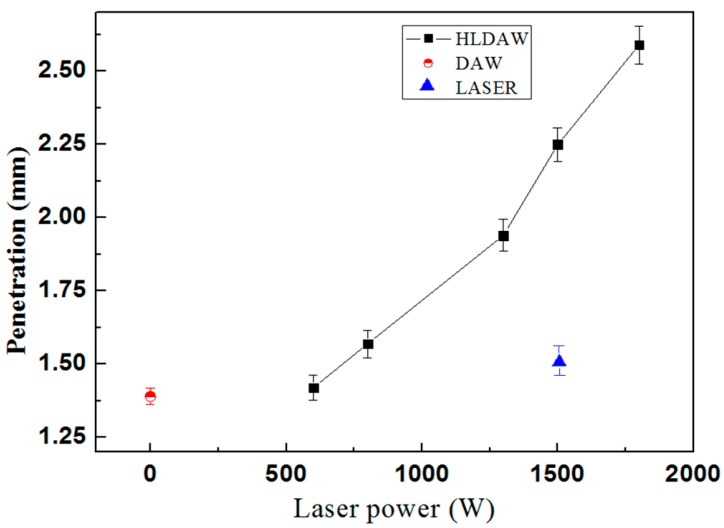
Penetration of DAW and HLDAW.

Figure 11 is the weld penetration comparison of pure laser welding, DAW and HLDAW with various laser powers. The weld penetration depth was improved in HLDAW, which was 1.8 times that of DAW and 1.5 times that of pure laser welding under the laser power of 1.5 kW. The weld penetration of HLDAW increased with laser power. Under the condition of this experiment, when the laser power was small, the energy density irradiated by the YAG laser onto the surface of the molten pool was relatively low, the synergy between laser and arc was weak and the temperature of molten metal in the middle of the molten pool was not significantly increased. Therefore, the melting depth of the HLDAW weld displayed the characteristics of the DAW weld. As the laser power increased beyond a certain threshold (600 W in this experiment), a mutually strengthening effect was achieved.

The higher the laser power, the higher the laser energy density at the laser point on the surface of the molten pool, which increases the temperature in the middle of the molten pool, resulting in the greater increase in the weld depth of HLDAW.

**Figure 12:**
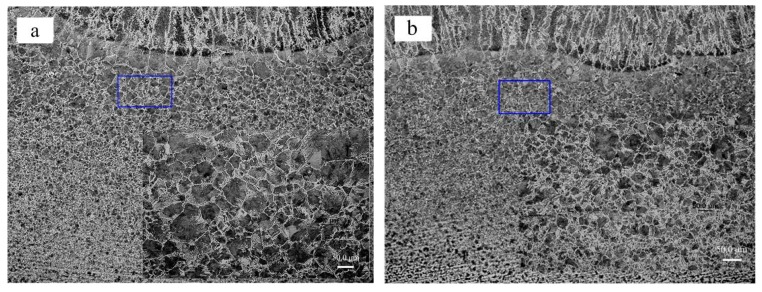
Microstructure of welded Q235B. (**a**) DAW(3#) υ = 5.0 mm/s, (**b**) HLDAW (18#) υ = 8.0 mm/s.

In addition to these differences in weld pool geometry, the microstructure of the weld with the same depth obtained by two welding methods (3# and 18#) is compared in Figure 12. Obtaining the same weld penetration, the line energy input of DAW was 682 J/mm, while the line energy input of HLDAW was only 591 J/mm, which is reduced by 13%. It was found that the microstructure of the weld zone or heat-affected zone was basically the same, except for a little difference in grain size. The microstructure of this weld was composed of columnar proeutectoid ferrite with intergranular acicular ferrite and pearlite. Compared with DAW, the microstructure and quantity of the proeutectoid ferrite precipitated along grain boundaries were tiny, and the superheated grains in the heat-affected zone were relatively small in the HLDAW weld. This indicated that the weld microstructure obtained by HLDAW is finer than DAW.

## 4. Conclusions

Based on the present study, the physical characteristics of coupled plasma and its influence on weld formation in HLDAW have been investigated. The following conclusions were drawn:

(1) The laser beam has significant influence on the shape, characteristic and energy distribution of the arc, and at the same time, the energy and transmission characteristics of this arc will be changed to some extent under the effect of the laser beam, which will modify the energy coupling, temperature distribution, fluid flow behavior and further influence the welding process.

(2) The experimental results showed that the electron density in the heat source center of HLDAW was 1.25 times that of DAW. The high concentration of charged particles provided a conductive channel for the two arcs, so as to restrain the drifting of arc roots during high-speed welding and obtain a more stable welding process.

(3) The reaction force produced by vapor ejected upward from the keyhole facilitates the molten metal flowing toward the bottom of the molten pool. The flowing from the periphery to the center of the pool enhanced the heat transfer to the bottom of the pool, which was helpful for the high temperature and superheated liquid metal to move downward the bottom of the pool and increase the weld depth. The preheating of the arc also intensifies the “digging” effect of the laser. The low-density arc dilutes the electron density of the laser plasma and reduces the absorption and reflection of laser energy by laser plasma to improve the penetrating effect of the laser beam. The above factors lead to the different flow modes of HLDAW from DAW, and obtain the greater weld depth.

(4) The experiment results showed that obtaining the same melting depth (1.39 mm), the line energy input of HLDAW was 13% lower than that of DAW, the heat affected zone is narrower and the grain size is smaller. The weld penetration depth was improved in HLDAW, which was 1.8 times that of DAW and 1.5 times that of pure laser welding under the laser power of 1.5 kW. The weld penetration of HLDAW increased with laser power.

## Figures and Tables

**Table 1:** Chemical components of Q235 steel and H08Mn2SiA welding wire (wt %).

C	Mn	Si	P	S	Cr	Ni	Cu	Fe
0.14–0.22	0.30–0.65	≤0.3	≤0.05	≤0.045	—	—	—	99.5–99.7
≤0.11	1.80–2.10	0.65–0.95	≤0.025	≤0.015	≤0.20	≤0.30	≤0.50	95.8–96.4

**Table 2:** Welding parameters.

Specimen Number	Laser Power (W)	Average Current (A)	Average Voltage (V)	Welding Speed (mm/s)
1#	1500	——	——	5
2#		——	——	5
3#	0	111.5	30.6	5
4#		110.8	30.2	5
5#		111.4	30.5	5
6#	600	113.2	31.1	5
7#		112.7	29.9	5
8#		113.3	29.4	5
9#	800	114.6	28.9	5
10#		114.1	28.7	5
11#		113.8	28.6	5
12#	1300	114.4	28.2	5
13#		115.2	28.2	5
14#		114.3	27.7	5
15#	1500	116.3	27.4	5
16#		116.8	27.3	5
17#		116.2	26.9	5
18#	1500	116.6	27.7	8
19#		116.3	27.4	8
20#	1800	118.4	26.7	5
21#		117.5	26.1	5
22#		117.1	25.9	5

**Table 3:** Parameters of the feature spectrum line [21].

λ/nm	*A_ki_*/s	*E_k_*/cm^−1^	*E_i_*/cm^−1^	*g_k_*
629.77927	6.12 × 10^4^	33,801.572	17,927.382	5
617.33352	2.31 × 10^5^	34,121.603	17,927.382	1
600.30119	1.79 × 10^5^	47,960.940	31,307.245	9
606.5482	1.07 × 10^6^	37,521.161	21,038.987	5

**Table 4:** Schematic of weld surface and section in DAW and HLDAW (P = 1.5 kW).

	Pure Laser(2#)	DAW(3#)	HLDAW(16#)
Weld appearance	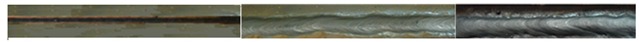		
Cross section